# Effect of an educational booklet for prevention and treatment of foot musculoskeletal dysfunctions in people with diabetic neuropathy: the FOotCAre (FOCA) trial II, a study protocol of a randomized controlled trial

**DOI:** 10.1186/s13063-020-4115-8

**Published:** 2020-02-13

**Authors:** E. Q. Silva, E. Y. Suda, D. P. Santos, J. L. Veríssimo, J. S. S. P. Ferreira, R. H. Cruvinel Júnior, R. L. Monteiro, C. D. Sartor, I. C. N. Sacco

**Affiliations:** 1 Department of Physical Therapy, Speech, and Occupational Therapy, School of Medicine, University of São Paulo, Rua Cipotânea, 51 - Cidade Universitária, São Paulo, São Paulo 05360-160 Brazil; 2 Department of Physical Therapy, Federal University of Amapá, Macapá, Amapá Brazil; 3 Department of Physical Therapy, Ibirapuera University, São Paulo, São Paulo Brazil

**Keywords:** Diabetic neuropathies, Exercise, Preventive care, Diabetic foot, Foot ulcer, Clinical trial, Physical therapy

## Abstract

**Background:**

This study is a part of a series of two clinical trials. We consider diabetic polyneuropathy (DPN), a common chronic and progressive complication of diabetes mellitus that has several impacts on individuals’ foot health and quality of life. Based on the current trends of self-monitoring and self-care, providing a tool with foot-related exercises and educational care may help patients to avoid or reduce the musculoskeletal complications resulting from DPN, improving autonomous performance in daily living tasks. The aim of this trial is to evaluate the effects of an educational booklet for foot care and foot muscle strengthening on DPN symptoms and severity, clinical outcomes, and gait biomechanics in patients with DPN.

**Methods/design:**

The FOotCAre (FOCA) trial II study has been designed as a single-blind, two-parallel-arm randomized controlled trial. It will include 48 patients with DPN who will be randomly allocated to a control (recommended foot care by international consensus with no foot exercises) group or an intervention (foot-related exercises using an educational booklet three times/week at home for 8 weeks) group. Participants from both groups will be assessed at baseline, after 8 weeks, and at 16 weeks for follow-up. The primary outcomes are the DPN symptoms and severity, and the secondary outcomes are foot–ankle kinematics, gait kinetics, plantar pressure distribution during gait, tactile and vibratory sensitivities, foot strength, functional balance, and foot health and functionality.

**Discussion:**

The booklet is a management tool that allows users to be autonomous in their treatment by choosing how and where to perform the exercises. This allows the patients to perform the exercises regularly as a continuous habit for foot care and health, which is an important element in the management of the diabetic foot. As the booklet focuses on specific foot–ankle exercises, we expect that it will improve the clinical aspects of DPN and produce beneficial biomechanical changes during gait, becoming a powerful self-management tool that can be easily implemented to improve the performance of daily living tasks.

**Trial registration:**

ClinicalTrials.gov, NCT04008745. Registered on 2 July 2019.

## Background

It is estimated that 438 million people worldwide will have diabetes mellitus (DM) by 2045 [1]. According to the National Institute of Diabetes and Digestive and Kidney Diseases, complications arising from DM have grown rapidly, with diabetic peripheral neuropathy (DPN) being the most common chronic complication, affecting up to 70% of the population with DM [2]. This complication affects the integrity of neural structures, starting with the peripheral sensitive nerves and progressing to the motor and autonomic nerves. DPN results in progressive loss of vibratory, thermal, tactile, and proprioception sensitivities [3], axonal dysfunction, and loss of motor units and motor axons [4–6].

DPN also impairs the structures of the musculoskeletal system, e.g., reducing the foot–ankle extrinsic and intrinsic muscle strength [7–11], increasing the proportion of fat tissue within foot muscles [2, 7], and reducing the mechanical properties of the calcaneal tendon [12]. It also alters the mechanics of locomotion, represented by impairments in the lower limbs’ muscle activation magnitude and timing [13–16], decreased ankle range of motion (ROM) [10, 17], and reductions in the ankle and increases in the hip moments [17–19]. Afferent (sensorial) and efferent (motor) impairments, such as the ones described, are the factors responsible for the gait dynamics alterations usually observed in this population [19]; these alterations change the plantar pressure distribution during gait [17, 20, 21], increasing the peak pressure and ultimately the risk of ulcer formation [22–24]. Due to the scenario described, it is expected that the incidence of plantar ulcers will escalate if therapeutic interventions aimed at improving and preventing the consequences of DPN are not implemented widely and prospectively.

There is evidence that foot–ankle exercises improve DPN symptoms. Sartor [13, 25] applied a therapeutic foot–ankle exercise program in patients with DPN for 12 weeks that included ROM and strengthening exercises, which resulted in improvements of DPN symptoms with a medium effect size. Kanchanasamut [26] showed that performing weight-bearing exercises for 8 weeks improved vibration perception. Chang [27] showed improvements in DPN symptoms after a 1-year intervention with a Buerger exercise-based program that focused on increasing foot and leg circulation. In addition to the evidence that foot–ankle exercises improve DPN symptoms, studies have also shown that these types of foot-related exercises can change the foot–ankle mechanics, reducing pressure-related variables while walking [13, 26, 28–33], increasing foot strength and function [31, 34, 35], and improving foot–ankle joint mobility [26, 33, 35–38].

Although it is clear that foot-related exercises are beneficial for people with DPN [26, 28, 31, 33, 34, 39–42], it has been shown that not all positive effects are retained at follow-up [31, 37], suggesting the need for self-management and continuous autonomous care to maintain the achieved benefits after intervention. One modality of self-management that has been shown to be effective for people with DM is structured education. Lincoln [43] showed the positive impact of a single 1-h educational session for improving foot-care behavior after a 1 year follow-up. Liang [44] also showed improvements in foot-care behavior after providing a foot-care kit and education to patients and caregivers. Thus, the use of structured education with the goal of encouraging DPN patients to perform foot–ankle exercises as part of their self-care habits may be beneficial in the management of DPN consequences and the prevention of further complications.

We note that only a few studies have attempted to assess the effects of self-management strategies and the autonomous practice of foot–ankle exercises for improving clinical and biomechanical outcomes in patients with DPN. Cerrahoglu [33] taught DM patients with and without DPN about foot–ankle exercises and called them weekly to increase adherence. Those strategies showed positive results, such as increased foot–ankle and first metatarsal joint ROM and decreased peak pressure during gait.

The use of educational materials can stimulate the incorporation of new care habits in the management of diseases. A lack of information is a major negative factor for patients’ compliance to treatments and recommendations. Most information given by health professionals during appointments is not retained by patients [45]. Therefore, the use of educational materials, such as booklets, that provide orientations in a structured and illustrated way can help enhance patients’ knowledge of the disease and its management. Such materials may promote the incorporation of foot–ankle exercise practices as a self-management strategy for patients with DPN.

The primary aim of this superiority, single-blind, two-parallel-arm, randomized controlled trial (RCT), the FOotCAre (FOCA) trial II, is to investigate the effects of an educational booklet that includes information about foot care and DPN consequences as well as an 8-week foot-related exercise program on DPN symptoms and severity. The secondary aims are to investigate the effects of this intervention at 8 and 16 weeks on tactile and vibration sensitivities, foot health and functionality, foot muscle strength, functional balance, plantar pressure, and foot–ankle biomechanics during gait.

Our first hypothesis (H1) is that the educational booklet, which includes information about foot care, DPN consequences, and foot-related exercise, will improve clinical aspects of DPN, reduce the symptoms of DPN, reduce the severity of DPN, improve the perception of tactile and vibratory stimuli on the foot, increase the strength of the foot muscles, increase the functional balance score, and improve foot health and functionality status.

The other hypothesis (H2) is that use of the booklet will produce beneficial biomechanical changes during gait. Thus, we expect its use to promote a more physiological foot rollover with a redistribution of plantar pressures during gait by either reducing peak pressure over risky areas or increasing it in dysfunctional plantar areas as well as increasing foot–ankle mobility and producing beneficial biomechanical changes during gait, such as (1) increasing the sagittal ankle ROM during the stance phase, (2) increasing the ankle dorsiflexion at the heel strike, (3) increasing the hindfoot to forefoot rotation ROM, (4) increasing the transverse plane ROM between the first and second as well as the second and fifth metatarsal bones, (5) increasing the ankle plantar flexion at the final phase of push-off, (6) increasing the ankle flexor moment and eccentric power at the heel strike phase, (7) increasing the ankle extensor moment and power at approximately 80% of the stance phase (watts/kilogram) corresponding to the propulsion phase, and (8) decreasing the deformation of the medial longitudinal arch angle at the midstance and push-off phases.

The present proposal innovates the use of a new paradigm that focuses on the autonomous and independent use of structured education and a foot-related exercise program to improve self-care and management with the aim of enhancing compliance to preventive strategies in people with DPN.

## Methods/design

### Trial design

This study is part of a series of two clinical trials: the FOCA trial I (SOPeD intervention) and FOCA trial II (booklet intervention). The FOCA trial II is a superiority, single-blind RCT with two parallel arms, in which participants with DPN will be randomly allocated to either a control group (CG), who will not receive any specific intervention besides the treatment recommended by a healthcare professional, or an intervention group (IG), who will perform foot-related exercises included in the booklet. This trial will have an allocation ratio of 1:1.

People with DM and DPN will be recruited from the Department of Endocrinology of the Hospital das Clínicas of the School of Medicine of the University of São Paulo and referred to a physical therapist who will perform the group allocation. The participants will then be referred to another physical therapist who will perform the initial blind assessment. Patients in the CG will not receive any specific intervention beyond usual care, including treatment recommended by the medical team, pharmacological treatment, self-care guidelines, and a weekly telephone call to check on the adherence to care, which will be maintained in both groups [46]. Patients in the IG will perform a foot-related exercise program included in an educational booklet three times/week at home for 8 weeks. After 8 weeks, the IG participants will be encouraged to continue this exercise until the end of the study, following the same schedule set in the intervention period. If proven effective, the benefits of the foot-related exercise protocol will be explained and offered to all control participants at the end of the study.

Patients from both groups will be evaluated for all outcomes at baseline (T0), 8 weeks (T8, end of intervention), and 16 weeks (T16, follow-up).

The Consolidated Standards of Reporting Trials (CONSORT) guidelines [47] will be followed. The study was approved by a research ethics committee (CAAE: 90331718.4.0000.0065) and was registered at ClinicalTrials.gov on July 2, 2019 (identifier NCT04008745).

### Study setting

The assessments will be performed at the Laboratory of Biomechanics of Human Movement and Posture (LaBiMPH) at the Department of Physical Therapy, Speech, and Occupational Therapy of the School of Medicine of the University of São Paulo, São Paulo, Brazil. The participants allocated to the IG will be treated at their homes, but the first session will take place at the outpatient clinic of the LaBiMPH. This first session will be conducted by a physical therapist, who will teach and supervise the correct execution of the exercises performed while using the booklet. All assessments will be performed at the same laboratory.

### Participants and recruitment

This study is currently recruiting patients (study start date: May 1, 2019) with a medical diagnosis of DM and DPN from the Department of Endocrinology of the Hospital das Clínicas of the School of Medicine of the University of São Paulo. Forty-eight participants with DPN will be recruited. The potential subjects will be interviewed by telephone and, once selected, assessed in the laboratory to confirm all eligibility criteria. This first laboratory assessment will represent the baseline condition (blind assessment).

### Eligibility criteria

#### Inclusion criteria

Adults (18 to 65 years), either sex, diagnosed with DM type 1 or 2 with at least mild DPN confirmed by fuzzy software (score ≥ 2) [13], who are able to walk independently and who do not have more than one amputated toe (that is not the hallux) will be eligible for inclusion in the study.

#### Exclusion criteria

Participants will be excluded from the study if they have any of the following criteria: an ulcer not healed for at least 6 months and/or an active ulcer; a history of surgical procedure to the knee, ankle, or hip or indication of surgery throughout the intervention period; arthroplasty and/or orthosis of lower limbs or indication of lower limb arthroplasty throughout the intervention period; diagnosis of other neurological disease outside of DM consequences; dementia or inability to give consistent information; have received any physiotherapy or offloading devices throughout the intervention period; have major vascular complications and/or severe retinopathy; or have a score of 12–21 (probable depression) on the Hospital Anxiety and Depression Scale (HADS).

### Procedure

**Fig. 1:**
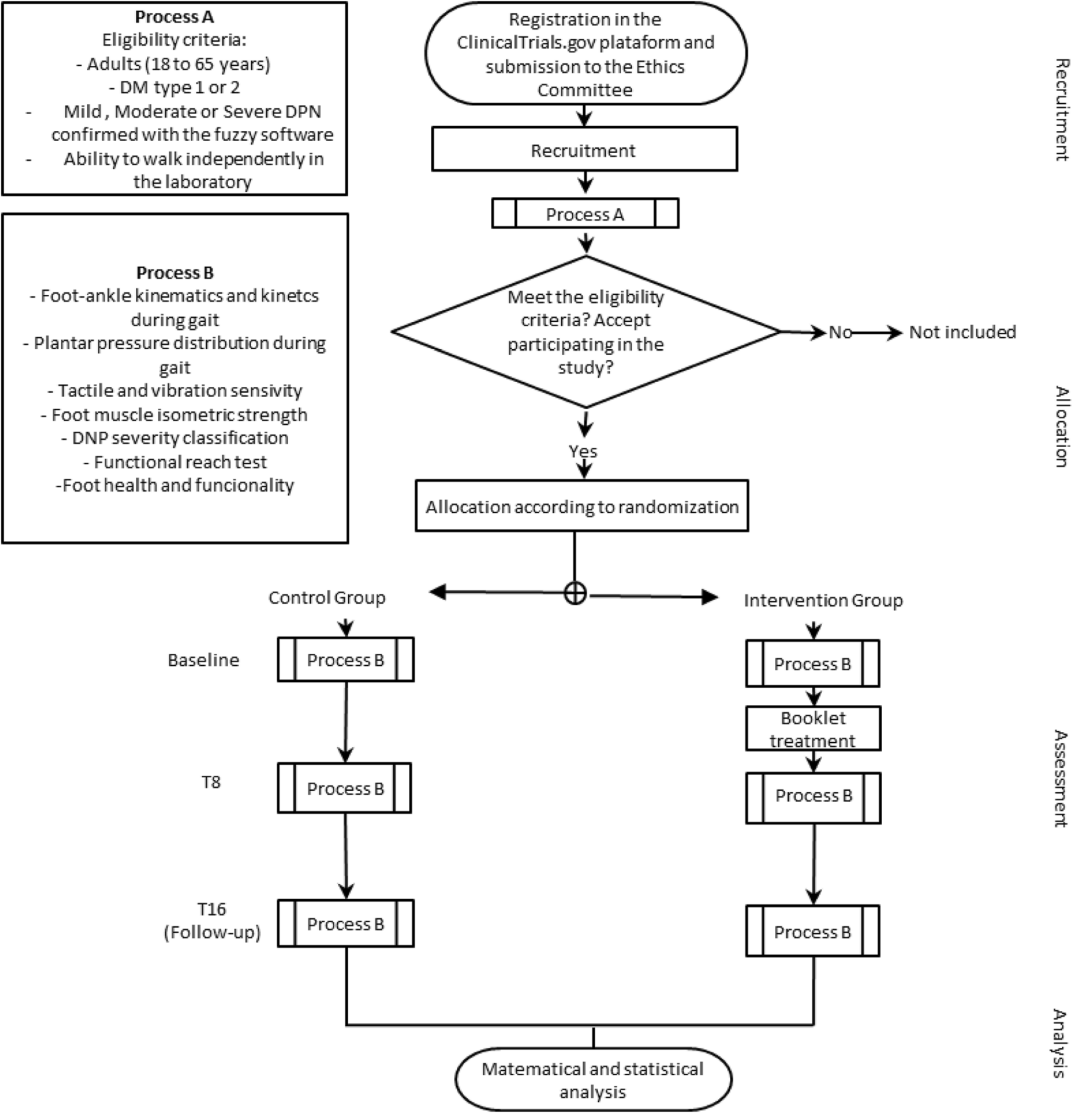
Consolidated Standards of Reporting Trials (CONSORT) flowchart illustrating the process of the FOCA trial II

The trial protocol follows all recommendations established by the Standard Protocol Items: Recommendations for Interventional Trials (SPIRIT) 2013 guidelines [48] (the checklist is provided in Additional file 1). Figure 1 presents the design and flowchart of the protocol according to the CONSORT guidelines [47].

### Randomization, allocation, and blinding

The randomization schedule will be prepared using Clinstat software (University of York, York, UK) [49] by an independent researcher (Researcher 1) unaware of the numeric codes for the CG and IG groups. The numerical sequence will be kept in opaque envelopes, numbered sequentially, following an order generated by the software. The randomization procedure will follow the instructions of [50]. This sequence will be kept private and stored in a location inaccessible to the blind assessors.

Potential patients will be assessed through an initial screening that consists of checking the eligibility criteria, classifying the DPN severity, and identifying those with a lower probability of adherence to the intervention due to depression. After receiving a patient’s informed consent to participate, the random allocation to either the IG or CG will be made by another independent researcher (Researcher 2), who will also be unaware of the codes. The envelope with the initially generated numerical sequence will then be opened, signed, and dated by Researcher 2, who will make the allocation. Only the main researcher (Researcher 3) responsible for intervention training will know the group allocation of participants. Patients are aware of the treatment and are thus not blind to the allocation. Researcher 3 will also be responsible for monitoring the intervention through weekly telephone calls and by checking the table in the booklet monthly. All patients’ personal data will be kept confidential before, during, and after the study by encoding participants’ names. Only Researcher 3 and the person receiving treatment will be aware of the meaning of each code. Patients will be allocated to study groups a maximum of 1 week after baseline evaluation.

Two other researchers (Researchers 4 and 5), who will also be blind to treatment allocation, will be responsible for all clinical, functional, and biomechanical outcome assessments.

To guarantee the blindness of the researchers, before each evaluation, patients will be instructed not to reveal whether they are in the CG or IG; their questions should be directed only for the main researcher (Researcher 3). All researchers will be blind to the block size used in the randomization procedure. The trial statistician will also be blind to treatment allocation until the main treatment analysis has been completed. The trial has an open label design, where only the outcome assessors are blind, so unblinding will not occur.

### Trial arms

#### Control group

The CG patients will not receive any specific intervention other than the treatment recommended by the healthcare team, which will include pharmacological treatment and self-care guidelines following the International Working Group on the Diabetic Foot guide [51]. These self-care guidelines have been adjusted for our setting in São Paulo and include (1) instructing patients to inspect their feet and the inside of shoes daily, wash feet daily (with careful drying, particularly between toes), avoid using chemical agents or plasters to remove calluses or corns, avoid cutting calluses or blisters without supervision, use emollients to lubricate dry skin, and cut toe nails straight across; (2) instructing patients to use socks without elastic and sewing; (3) instructing patients to avoid walking barefoot or wearing shoes without socks or slippers and to seek medical assistance whenever identifying problems in their feet; and (4) providing education aimed at improving foot-care knowledge and behavior as well as encouraging the patient to adhere to this foot-care advice. These guidelines will be maintained for both groups. Patients in this group will also receive a weekly telephone call to check the adherence to the care recommended by the medical staff and the foot-care guidelines and to avoid the nocebo effect.

#### Intervention group

Patients in the IG will receive an educational booklet with two parts. The first part includes educational information to guide the individuals to change their health behavior regarding autonomous foot care, with information about DPN, footwear, and benefits of exercising the feet and ankles. The second part includes a home-based physiotherapeutic foot-related exercise program comprising six exercises to be performed three times per week at home for an 8-week period. This program will be supervised by Researcher 3 through weekly telephone calls and after the first in-person supervised session at the department. The patients will receive access and all instructions on how to use the tool on the first day. During the follow-up period, IG participants will be encouraged to follow the same schedule set by the project until the end of the study (16 weeks after allocation), but will not be weekly monitored, and will be encouraged to continue exercising in the future.

### Outcomes and measures

#### Participant timeline

Two researchers (Researchers 4 and 5) who are blinded to group allocation will perform all assessments. Participants of both groups will be assessed at baseline (T0), at the end of intervention (T8, 8 weeks after baseline), and at follow-up (T16, 16 weeks after baseline). Table 1 shows the schedule of enrollment, interventions, and assessments according to the SPIRIT guidelines [48].

**Table 1:**
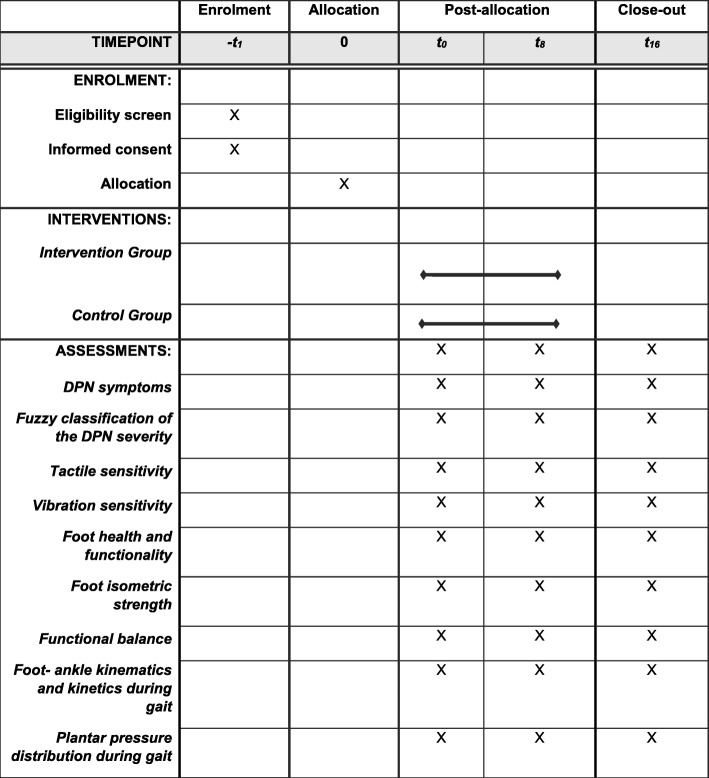
Schedule of enrollment, interventions, and assessments of the FOCA trial II, following SPIRIT guidelines

#### Screening measures

An initial anamnesis will be performed to check the eligibility criteria, including clinical, anthropometric, and demographic characteristics of all participants. The classification of DPN severity will be made using the fuzzy score from a web software program [13, 52]. Participants with scores equal to or above 2.0, corresponding to mild DPN, will be included in the study. Those who score between 12 and 21 (probable depression) on the Brazilian-Portuguese HADS will not be included [53].

### Measures of primary and secondary outcomes

The DPN symptoms and the classification of the DPN severity will be the primary outcomes. The foot–ankle kinematics and kinetics during gait, plantar pressure distribution during gait, tactile and vibration sensitivities, foot health and functionality, foot strength, and functional balance will be the secondary outcomes.

#### DPN symptoms

Patients will answer the Brazilian version of the Michigan Neuropathy Screening Instrument (MNSI) [54]. This questionnaire has 15 questions about the sensitivity of the feet and legs and is self-administered. A score of 1 point is given for answers of “yes” for questions 1, 2, 3, 5, 6, 8, 9, 11, 12, 14, and 15 and “no” for questions 7 and 13. Questions 4 and 10 evaluate circulatory deficits and general asthenia, respectively, and neither is included in the final score. The sum of all scores ranges from 0 to 13, and the larger the score, the worse the DPN.

#### Fuzzy classification of the DPN severity

The classification of the DPN severity will be made using the Decision Support System for Classification of Diabetic Polyneuropathy [13, 52] developed by the LaBiMPH and publicly available at http://www.usp.br/labimph/fuzzy/. This decision will be based on fuzzy logic with the input variables of signs and symptoms extracted from the MNSI as well as tactile sensitivity (through the number of non-touch areas using a 10-g monofilament) and vibration sensitivity (measured by vibrating a tuning fork at 128 Hz), characterized as absent, present, or diminished. The software gives a score from 0 to 10, with higher scores indicating more severe DPN.

#### Tactile sensitivity

Tactile sensory deficits will be assessed by a 10-g monofilament [46] in four plantar areas (plantar surface of the hallux and heads of the first, third, and fifth metatarsals). This instrument has good reliability and validity in elderly individuals [55]. The monofilament will be applied perpendicularly to the skin surface three times on the tested areas with sufficient force to cause the filament to bend or buckle. The sequence of the tested areas will be randomized. The patient will not be able to see the monofilament or where it is being applied. The number of areas in which the patient does not feel pressure will be recorded [56]. The greater the number of areas marked, the greater the impairment of tactile sensitivity.

#### Vibration sensitivity

Vibration testing will be conducted with the timed method using a 128-Hz tuning fork applied to the dorsal surface of the distal phalanx of the hallux. The time (in seconds) at which vibration sensation diminishes beyond the examiner’s perception will then be recorded from both sides on a standardized form [57]. Values less than 10 s will be classified as present vibratory sensitivity, those greater than 10 s will be classified as decreased vibratory sensitivity, and no perception will be classified as absent vibratory sensitivity.

#### Foot health and functionality

The Brazilian-Portuguese version of the Foot Health Status Questionnaire (FHSQ-BR), which has been translated and validated [58], will be used. This questionnaire is divided into three domains; we will use Domains I and II. These domains comprise questions with answer options presented in affirmative sentences and corresponding numbers. Domain I evaluates foot health in four dimensions: foot pain, foot function, footwear, and general foot health. Domain II evaluates the general state of health, also in four dimensions: general health, physical activity, social capacity, and vitality. Domain III collects general demographic data. Domains I and II have a score from 0 to 100 points, where 100 is the best condition and 0 is the worst. The scores will be calculated using the FHSQ software version 1.03 (Care Quest, Australia).

#### Functional balance measure

The functional balance assessment will be performed according to [59] using the Functional Reach Test (FRT). Subjects will be asked to assume a standing position without shoes or socks. Patients will be asked to stand with their shoulders perpendicular to the reach measurement device (measuring tape), which will be attached to the wall and parallel to the floor at the height of the patient’s acromia. The upper extremities should not contact the wall during the task. In order to maintain identical foot placement during all testing conditions, the foot position will be traced on a sheet attached to the surface of the floor. The initial measurement (Position 1) will correspond to the position of the third metacarpal at the beginning of the measuring tape; the end of the measurement is where the third metacarpal has reached on the measuring tape after a forward movement (Position 2). The patient will be instructed to lean forward as much as possible without losing balance, flexing the hips, or taking a step. Functional reach is defined as the mean difference between Positions 1 and 2. Three trials will be performed, and the average score will be used for statistical purposes. The greater the distance achieved, the better the functional balance.

#### Foot muscle isometric strength

The isometric strength of the flexor muscles of the hallux and lesser toes will be measured according to Mickle [60] using a pressure platform (emed q-100; Novel GmbH, Munich, Germany). Subjects will stand and push down on the platform as hard as possible with the toes and hallux, controlling for excessive body sway. The plantar regions corresponding to the hallux and the toes will be identified by a standard mask from Novel-win Multimask software v.9.35 (Novel GmbH). The average of three trials on each foot (left and right) will be used for statistical purposes. The outcomes will be the maximum force under the hallux and toes, normalized by bodyweight.

#### Foot–ankle kinematics and kinetics during gait

Gait kinematics will be assessed by three-dimensional (3D) displacements of passive reflective markers (9.5 mm in diameter) tracked by eight infrared cameras at 100 Hz (VERO, Vicon Motion Systems Ltd., Oxford Metrics, Yarnton, UK) and the NEXUS 2.8 motion capture software (Vicon Motion Systems Ltd.). Forty-two markers will be placed on both limbs of the subject (pelvis, thigh, leg, ankle, and foot) according to the Plug-In Gait and Oxford Foot Model [61] setup protocols. The laboratory coordinate system will be established at one corner of the force plate, and all initial calculations will be based on it. Each lower limb segment (shank and thigh) will be modeled based on surface markers as a rigid body with a local coordinate system that coincides with the anatomical axes. Translations and rotations of each segment will be reported relative to the neutral positions defined during the initial static standing trial. All joints will be considered to be spherical (i.e., with three rotational degrees of freedom). Ground reaction forces will be acquired by a force plate (OR-6-1000, AMTI, Watertown, MA, USA) embedded in the center of a 10-m walkway at 1 kHz. Force and kinematic data acquisition will be synchronized and sampled by an analog-to-digital (A/D) board (Control Box LOCK, 192 kHz, 24 bits; Vicon).

Participants will be asked to walk at a comfortable, self-selected speed, with a maximum variation of 5% between measurements, thus ensuring that the same speed is maintained in all assessments (T0, T8, and T16). After a complete habituation to the laboratory environment, 10 valid steps will be acquired on each side during gait.

The automatic digitizing process, 3D reconstruction of the markers’ positions, and filtering of kinematic data will be performed using the NEXUS software. Kinematic data will be processed using a zero-lag second-order low-pass filter with a cutoff frequency of 6 Hz. Ground reaction force data will be processed using a zero-lag low-pass Butterworth fourth-order filter with a cutoff frequency of 50 Hz.

The bottom-up inverse dynamics method will be used to calculate the ankle moments in the sagittal plane. For the calculation of ankle power, the calculated joint moment and the ankle angular velocity in the sagittal plane will be considered. Calculation of all discrete variables from the time series obtained will be performed using a custom-written MATLAB function (MathWorks, Natick, MA, USA).

The following kinematic variables will be analyzed for the stance phase: (1) sagittal ankle ROM, (2) ankle dorsiflexion at heel strike, (3) ankle plantar flexion at push-off, (4) hindfoot to forefoot rotation ROM, (5) transverse plane ROM between first and second metatarsal bones and between second and fifth metatarsal bones, and (6) deformation of the longitudinal medial arch. The ankle kinetic variables to be analyzed are (1) flexor moment and eccentric power at heel strike and (2) extensor moment and power at approximately 80% of stance phase, corresponding to the propulsion phase.

#### Plantar pressure distribution during gait

A 700 × 403 × 15.5-mm pressure platform (emed q-100; Novel GmbH) with 6080 sensors (four sensors per square centimeter) will be used to acquire plantar pressure data during gait at 100 Hz. Participants will walk barefoot three times over the platform with a self-selected gait speed (the same as in the kinematic trials), covering a distance of 4 m. Both feet will be analyzed for each patient. Based on the algorithm by Giacomozzi [62], peak pressure, contact area, and pressure–time integral in seven anatomical plantar regions—heel, midfoot, medial forefoot, medium forefoot, lateral forefoot, hallux, and toes—will be analyzed. This method relies on the integration of a 3D motion capture system (Vicon system), a pressure measurement device (emed q-100), a multi-segment foot model, and an algorithm to identify regions of interest.

### Intervention

Participants allocated to the IG will receive an educational booklet with two parts. The first part includes educational information to guide the individuals to change their health behavior regarding autonomous foot care, with information about DPN, footwear, and benefits of exercising the feet and ankles. The second part includes a home-based physiotherapeutic foot-related exercise protocol comprising six exercises.

Before starting the exercise protocol, the patients will be instructed by the main researcher on how to perform the exercises using the booklet. The first session will be supervised at the outpatient clinic of the LaBiMPH, providing a reliable therapeutic environment for the first intervention.

The exercise program includes strengthening of the intrinsic foot muscles and the extrinsic foot–ankle muscles, and it consists of the following steps:

1. Warm-up: the patients will warm up the feet and ankles with three exercises. They will be instructed to massage their feet, and then use a spiky ball to do a deep tissue massage, and subsequently perform rotating movements in each toe, one by one. Altogether, these exercises should be performed within 2–3 min.
2. A total of six exercises will be performed: four exercises for the intrinsic foot muscles and two for the extrinsic foot–ankle muscles. The exercises will be performed in the order suggested in the booklet using objects such as cotton, pencil, balls, and chairs. The interphalangeal, metatarsophalangeal, and ankle joints are targeted in the protocol. The following muscle groups are targeted in the protocol: medial-plantar aspect (abductor hallucis, flexor hallucis brevis, and adductor hallucis), lateral-plantar aspect (abductor digiti minimi, flexor digiti minimi brevis, and opponens digiti minimi), middle-plantar aspect (flexor digitorum brevis, quadratus plantae, lumbrical muscles, plantar interosseous muscles, and dorsal interosseous muscles), and dorsal aspect (extensor digitorum brevis and extensor hallucis brevis).

The foot-related exercises will first be performed in the sitting position in one set with 30 repetitions. If the patient finds this too easy, the exercise will then be performed in the standing position, and then standing on one foot. The patient can also increase the number of sets. Patients will follow the exercise program from the booklet with instructions for each exercise prescribed, and after each task, they will fill out a table stating the perceived effort for each exercise (using a Likert scale). At the end of each exercise, patients will define their effort using a visual analog scale (VAS). If the effort is between 0 and 5, the individual should progress to the next level of the exercise (e.g., from sitting to standing, or using a different object) for the next session. If the effort is between 6 and 8, the same volume and level of difficulty should be maintained. If the effort is between 9 and 10, the amount of repetitions should be decreased or the position in which the exercise is performed should be modified (e.g., from standing to sitting) (see Additional file 2).

Patients will follow this regime three times a week for 8 weeks, for a total of 24 sessions. The duration of a session should be no longer than 30 min. The perceived effort scale will be used to regulate each patient’s individual effort for his/her progression to the next session of exercises, and it will be recorded by the participant using the monthly table in the booklet (see Additional file 2). The supervision will occur weekly via telephone calls and monthly by Researcher 3 checking the table in the booklet.

The discontinuation criteria for the exercises include cramps, moderate to intense pain, fatigue, or any other condition that exposes the patient to any discomfort. The other discontinuation criterion for the intervention is the occurrence of a foot ulcer as assessed by a blinded podiatrist nurse specializing in diabetic feet. The patients will be advised to report any sign of tissue damage to Researcher 3.

### Data management

The study steering committee comprises two Ph.D. students (blind evaluators), two master’s students (responsible for data collection), two undergraduate students (responsible for data tabulation and codification), a coordinator (responsible for managing the project), and an assistant researcher (responsible for the recruitment and scheduling of collections).

All information collected during the protocol will be entered into an electronic form by those responsible for data collection. The integrity and validity of the data will be verified at the time of data entry (edit checks). Identification of potential recruits will be done by the project manager and the research assistant. The research assistant will be trained on how to approach the eligible subjects during the initial recruitment contact for the survey (made by phone calls) and how and when to contact them for follow-up and data collection.

### Oversight and monitoring

The data monitoring committee (steering committee) and the Faculdade de Medicina da Universidade de São Paulo Board will regularly monitor (depending on the recruitment numbers and collections performed) the study datasets and make recommendations on necessary protocol modifications or termination of all or part of the study. A trimester meeting is held to facilitate the study development. All team members can request meetings as needed.

All adverse events occurring during the clinical trial period will be recorded. Minor adverse effects potentially expected are sore muscles and tiredness after performing the proposed exercises. The patients will be advised to report any discomfort and foot pre-ulcerative signs (blisters, calluses) or foot ulcers to Researcher 3, who will ask for the blinded podiatrist nurse to assist the patient.

### Sample size and statistical analysis

The sample size was calculated using the G*Power v.3.1 program [63] based on the following outcomes: MNSI DPN symptoms (primary) and ankle sagittal ROM during gait (secondary). These two outcomes were chosen because they reflect important functional gains for patients with DPN. Thus, two sample size calculations were performed, and we selected the one that resulted in the largest number of participants. The effect sizes used for both calculations were based on a study that evaluated the effect of 12 weeks of supervised foot exercise in patients with DM [34]. In that study, the improvement in the MNSI symptoms had a medium effect size (0.52), as did gains in the ankle sagittal ROM during gait (0.46). We chose to use half of the effect size obtained in [34], because the nature of our intervention is home-based, whereas Sartor’s was face to face, which is assumed to be more effective.

The input factors for sample size calculation resulted from an *F* test statistical design with interaction between and within factors with two repeated measures and two study groups, a statistical power of 0.80, an α of 0.05, and effect sizes of 0.26 (MNSI symptoms) and 0.23 (ankle ROM). The resulting sample sizes were 32 and 40 individuals for MNSI and ROM outcomes, respectively. Therefore, we defined our sample size as 40. Assuming a 20% dropout rate during the study, a sample size of 48 patients is needed.

The inferential statistical analyses will be based on using intention-to-treat and per protocol analyses. The missing data will be treated by imputation methods depending on the type: missing completely at random, missing at random, or not at random [64]. The per protocol analysis will include only those patients who complete follow-up in the allocated intervention group. If there is evidence that the difference in the treatment depends on certain patient characteristics identified in the baseline assessment, a subgroup analysis will be performed. Confirmation of normality (Kolmogorov–Smirnov test), homoscedasticity (Levene test), and imputation of the means for the missing data of variables with normal distribution will be conducted. After that, mixed general linear models of analysis of variance for repeated measures will be used to detect treatment–time interactions, followed by the Newman–Keuls post hoc test to obtain group effect (intervention and control), time effect (between T0, T8, and T16), and group–time interaction. Significant differences will be considered at α = 5%, but for the description of the effect of the intervention, the effect size (Cohen coefficient) and difference between the means will be calculated with their respective 95% confidence intervals.

## Discussion

We have presented the rationale and design of FOCA II, a single-blind RCT with parallel arms, on the efficacy of a self-managed foot–ankle exercise program in patients with DPN. This RCT will provide important data on education and self-management of the foot–ankle exercise effectiveness in reducing DPN symptoms and severity as well as the effects on gait biomechanics. The outcomes may contribute to increasing the benefits of foot-related exercises for this population and preventing several complications related to DM and DPN.

Studies with global exercise protocols with durations of 12 weeks or more have shown an increase in gait velocity [65] and joint ROM [31, 37] as well as improvements in the musculoskeletal condition [65]. Although few studies have evaluated the effect of specific foot–ankle exercises in patients with DM, there is evidence that improvements in DPN symptoms, in the structure of the musculoskeletal system, and in biomechanical variables during gait can be achieved with this kind of intervention [26–31, 34–38, 66].

However, it has been shown that not all beneficial effects are preserved after the end of the intervention [31, 37], demonstrating that self-management and continuous autonomous care are important to keep the achieved results after any intervention. One way of improving self-care is through patient education: a planned learning experience to improve patients’ knowledge and health behavior [67]. A systematic review [68] of self-care education programs in people with DM showed that this kind of strategy is favorable for all biopsychosocial and economic outcomes, but the authors reinforced the need for further studies due to the wide variety of methodologies and study variables.

The booklet proposed for this RCT is a print-based resource that contains information about DM and DPN (see Additional file 2). Therefore, this educational material can provide guidelines in a structured and illustrated way to promote the incorporation of foot–ankle exercises as a self-management behavior in patients with DPN. The booklet is a rehabilitation technology intended to promote independence and autonomy in the management of patients’ treatment by allowing them to choose when and where to perform the exercises. It also allows the user to perform the exercises in a progressive and individualized way as continuous musculoskeletal care, which is an important factor in the management of the diabetic foot. If our hypotheses that the use of the booklet for foot–ankle exercises improves the clinical aspects of DPN and produces beneficial biomechanical changes during gait are true, the booklet could be a powerful self-management tool that is easily implemented in public and private healthcare systems.

### Trial status

The trial was registered at ClinicalTrials.gov, with identifier NCT04008745, version 1, on 2 July 2019 and was last updated on the same date. Participant recruitment began on 1 May 2019 and is expected to continue until mid-2021. Randomization of the participants was performed on the same day.

## Supplementary information

**Additional file 1.** SPIRIT 2013 checklist: Recommended items to address in a clinical trial protocol and related documents.

**Additional file 2.** Exercise booklet.

## Data Availability

All personal data from potential or enrolled participants will be maintained confidential before, during, and after the trial by encoding the participants’ names. All data access and storage are in keeping with National Health and Medical Research Council guidelines, as approved. All files will be available from the database published at figshare.com. The main researcher will report all important protocol amendments to the investigators, review boards, and trial registration. Upon completion of the study, supported data will be available upon request.

## Abbreviations

CG: Control group
DM: Diabetes mellitus
DPN: Diabetic peripheral neuropathy
FHSQ-BR: Brazilian-Portuguese version of the Foot Health Status Questionnaire
FOCA: Foot Care (trial)
FRT: Functional Reach Test
HADS: Hospital Anxiety and Depression Scale
IDF: International Diabetes Federation
IG: Intervention group
IWGDF: International Working Group on the Diabetic Foot
LaBiMPH: Laboratory of Biomechanics of Human Movement and Posture
MNSI: Michigan Neuropathy Screening Instrument
RCT: Randomized controlled trial
ROM: Range of motion
T0: Baseline
T16: 16 weeks follow-up
T8: 8 weeks
