# Supplementary material for: Effect of an educational booklet for prevention and treatment of foot musculoskeletal dysfunctions in people with diabetic neuropathy: the FOotCAre (FOCA) trial II, a study protocol of a randomized controlled trial
Source: Trials. 2020 Feb 13;21:180. doi: 10.1186/s13063-020-4115-8 (PMC7020575; doi:10.1186/s13063-020-4115-8)
Supplement: Supplementary file 2 — Additional file 2. Exercise booklet. [file 13063_2020_4115_MOESM2_ESM.pdf]

Universidade de São Paulo

Cartilha de exercícios para pés e tornozelos destinado a pessoas com Diabetes Mellitus

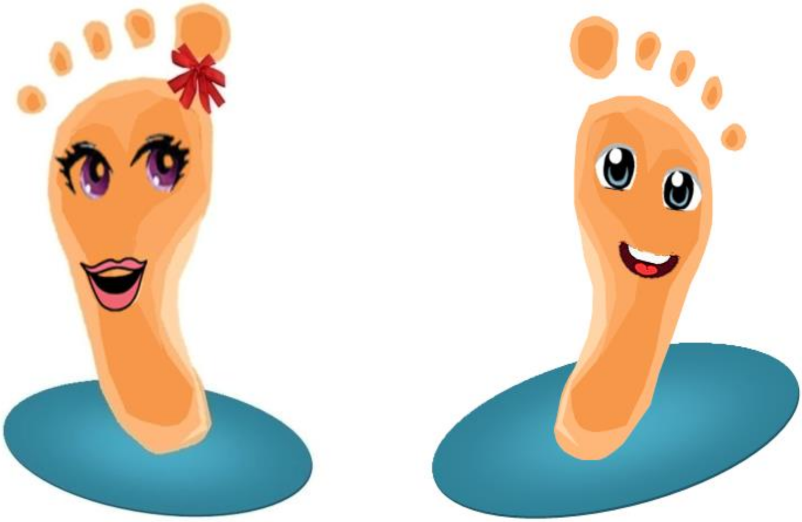

Laboratório de Biomecânica do Movimento e da Postura Humana

www.usp.br/labimph

EQUIPE:

Jady Luara Veríssimo

Profa. Dra. Cristina Dallemole Sartor

Profa. Dra. Isabel de Camargo Neves Sacco

Profa Dra. Maria Helena Morgani Almeida

Colaborador:

Gil Kioshi Konno

Projeto desenvolvido no Laboratório de Biomecânica do Movimento e Postura Humana – LaBiMPH - Departamento de Fisioterapia, Fonoaudiologia e Terapia Ocupacional – Faculdade de Medicina da Universidade de São Paulo.

www.usp.br/labimph

Sumário

Apresentação das personagens........................... 4

Introdução.. 5

Informações importantes.................................. 6

Como funciona a cartilha.................................. 8

Você possui outras dúvidas?............................... 14

Preparação dos pés. 16

Exercício 1... 18

Exercício 2... 20

Exercício 3... 22

Exercício 4... 24

Exercício 5... 26

Exercício 6.. 28

O que fazer quando terminar a tabela? 30

Alguns cuidados que devemos ter com os pés 31

Aos profissionais da saúde................................ 33

Apresentação das personagens

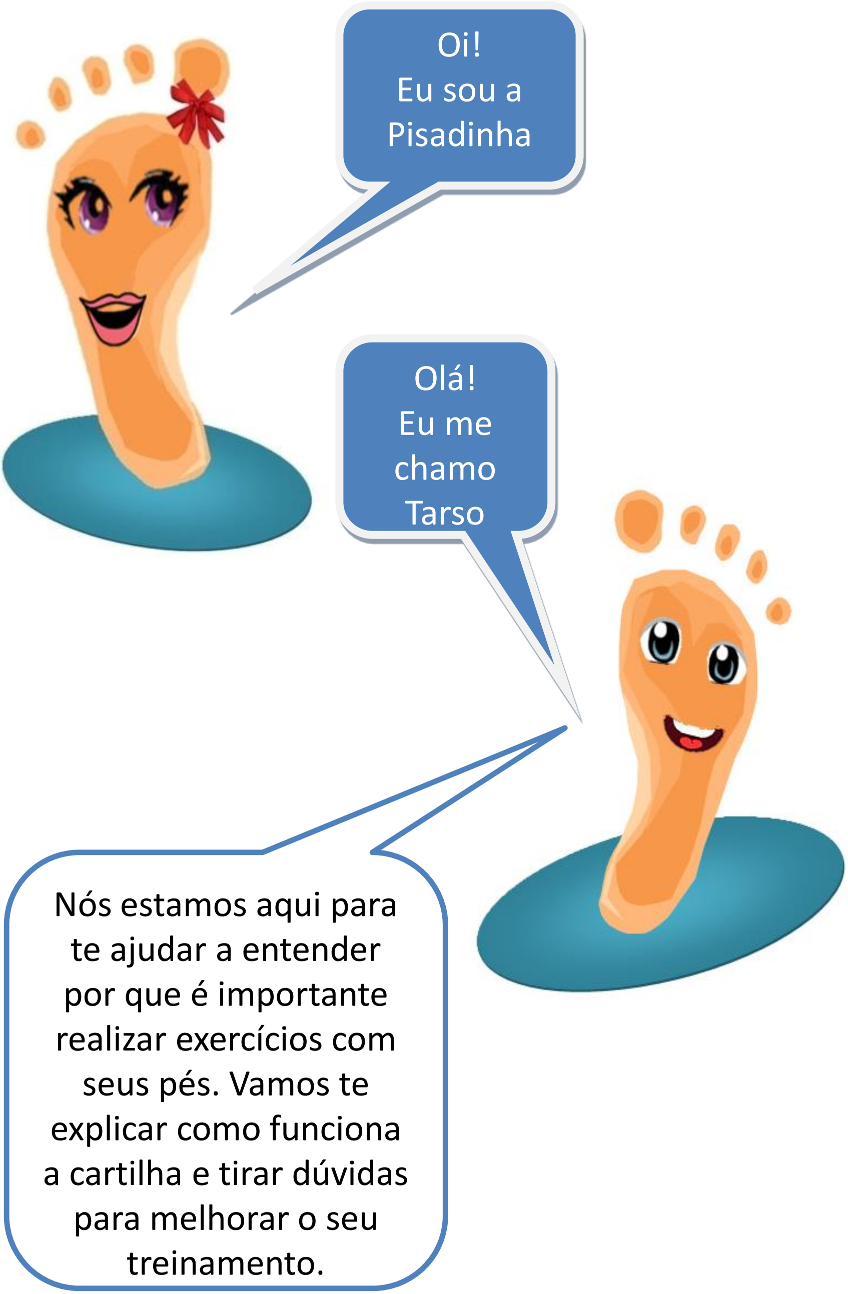

Introdução

Esta cartilha com ilustrações tem a intenção de fornecer orientações e incentivar a realização de exercícios específicos para os **pés e tornozelos** para pessoas com Diabetes. A cartilha também direciona a evolução na execução dos exercícios para favorecer o seu ganho de movimento e superação de novos desafios.

Neuropatia Diabética

A Neuropatia Diabética é causada por **aumento da quantidade de açúcar no sangue** (alto nível de glicemia). E com o passar do tempo essa doença pode provocar problemas em todo o corpo, mas principalmente nos pés. Essa doença pode levar à perda de força muscular e de movimentos nos pés e nas mãos.

Informações importantes:

Por que é importante fazer os exercícios?

Fazer exercícios específicos para os pés diariamente é uma maneira de diminuir alguns efeitos causados pelo Diabetes. Há melhora do movimento das articulações, melhora da função muscular, melhora da percepção do que está acontecendo com seus pés. Com isso, seu equilíbrio, seu jeito de andar e sua **confiança** em se movimentar aumentam. O Diabetes dificulta a realização de algumas atividades do dia a dia, como caminhar, subir e descer escadas, passar por obstáculos e superfícies instáveis, mas com os exercícios específicos essas dificuldades podem **diminuir e até desaparecer.**

O que devo sentir ao fazer os exercícios?

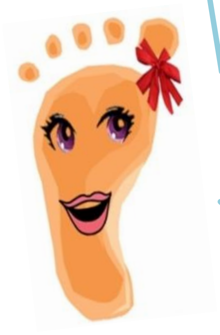

Você sentirá que os músculos de seus pés e pernas ficarão **cansados** durante a realização dos exercícios. Isso é **bom e desejável**! Ao exercitar os músculos, eles precisam ser desafiados para que, pouco a pouco, fiquem mais fortes e saudáveis.

Qualquer pessoa pode fazer esses exercícios?

Sim, qualquer pessoa com ou sem diabetes, tipo 1 ou 2, com ou sem neuropatia diabética pode realizar esses exercícios. Mas as pessoas que tiverem ferida aberta, úlcera, saída de líquido ou pus; tiver infecções ou dores intensas (ex: artrose, artrite reumatoide, etc), NÃO DEVERÃO FAZER OS EXERCÍCIOS e precisam procurar atendimento médico!

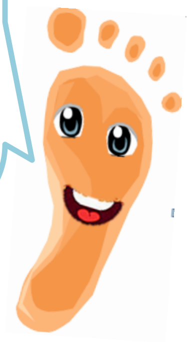

Como o Diabetes afeta os meus pés?

Controlar a diabetes é muito importante, pois o alto nível de glicemia, que é a **quantidade de açúcar no sangue,** pode afetar os nervos dos pés e causar a neuropatia diabética, que gera a sensação de formigamento e a perda de sensibilidade nessa região. Isso pode fazer com que você não sinta dor, pressão, alterações de temperatura ou até mesmo que **não perceba um machucado no pé,** o que pode gerar infecções aumentando o risco de amputações. Além disso, ocorre grande fraqueza muscular e as articulações ficam rígidas ("duras") e dificultam o movimento e o equilíbrio.

A Cartilha funciona assim:

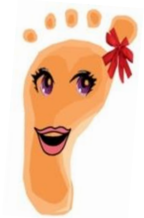

A cartilha é composta por vários exercícios e cada um deles tem o objetivo de trabalhar os grupos musculares dos pés e tornozelos. E para facilitar e acompanhar a sua evolução, a cartilha conta com foto, explicações e tabelas, que funcionam da seguinte maneira:

Passo 1 – observe a descrição de como deve ser feito o exercício, e as fotos que exemplificam o modo de excussão:

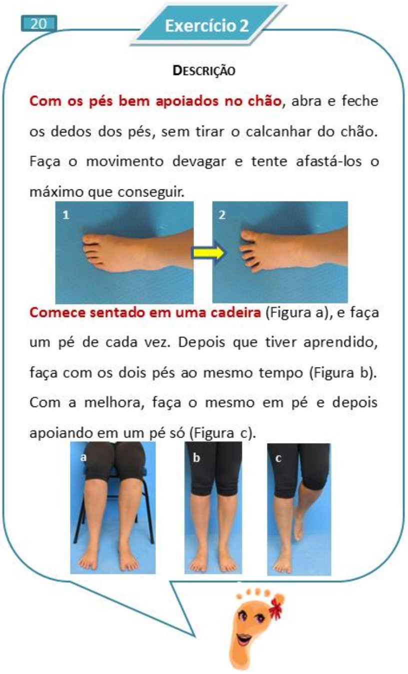
20

Exercício 2

DESCRIÇÃO

Com os pés bem apoiados no chão, abra e feche os dedos dos pés, sem tirar o calcanhar do chão. Faça o movimento devagar e tente afastá-los o máximo que conseguir.

1 2

Comece sentado em uma cadeira (Figura a), e faça um pé de cada vez. Depois que tiver aprendido, faça com os dois pés ao mesmo tempo (Figura b). Com a melhora, faça o mesmo em pé e depois apoiando em um pé só (Figura c).

a b c

Passo 2 – Há um calendário para cada exercício, que possui uma linha indicando a quanto dias está realizando os exercícios. Ou seja , pode se comparar o seu desempenho .

	Calendário da realização dos exercícios										
	Dias	1	2	3	4	5	6	7	8	9	10
	Data										
Sentado	30x										
	2x30										
Em pé	30x										
	2x30										
Sobre um pé	30x										
	2x30										

Passo 3- Há também um espaço na tabela destinado para colocar a data em que realizou os exercícios.

	Calendário da realização dos exercícios										
	Dias	1	2	3	4	5	6	7	8	9	10
	Data	02.9	03.9								
Sentado	30x										
	2x30										
Em pé	30x										
	2x30										
Sobre um pé	30x										
	2x30										

Passo 4 – Cada exercício possui uma progressão, como está indicado:

	Calendário da realização dos exercícios										
	Dias	1	2	3	4	5	6	7	8	9	10
	Data										
Sentado	30x										
	2x30										
Em pé	30x										
	2x30										
Sobre um pé	30x										
	2x30										

Por exemplo, 30x significa repetir o movimento 30 vezes; na linha de baixo "2x30" significa fazer o exercícios 30 vezes descansar um pouco **(aproximadamente 20 segundos)** e fazer o exercícios mais 30 vezes, ou seja, dar um intervalo curto entre as repetições.

Conforme, for progredindo nos exercícios, o modo execução também irá mudar (observe o retângulo vermelho).

	Calendário da realização dos exercícios										
	Dias	1	2	3	4	5	6	7	8	9	10
	Data										
Sentado	30x										
	2x30										
Em pé	30x										
	2x30										
Sobre um pé	30x										
	2x30										

Passo 5 – No calendário também há um espaço para anotar qual a sensação ao realizar o exercício. Por exemplo: A primeira vez que fez o exercício, achou **fácil** realiza-lo, marque um **"F"**, mas no segundo dia achou **difícil** realizar o exercício, então marque **"D"**(Observe a imagem indicadas pelas setas).

	Calendário da realização dos exercícios										
	Dias	1	2	3	4	5	6	7	8	9	10
	Data	02.9	03.9								
Sentado	30x	F									
	2x30		D								
Em pé	30x										
	2x30										
Sobre um pé	30x										
	2x30										

Definir como foi realizar o exercício é muito importante, pois é a partir dessa definição que é realizada a progressão.

Passo 6– Para classificar qual foi a sensação ao realizar os exercícios, utilizamos uma escala de 0 A 10, sendo que quanto mais próximo do 0 mais fácil foi realizar o exercício e quando mais próximos do 10 mais difícil foi realiza-lo.

Passo 6– Para classificar qual foi a sensação ao realizar os exercícios, utilizamos uma escala de 0 A 10, sendo que quanto mais próximo do 0 mais fácil foi realizar o exercício e quando mais próximos do 10 mais difícil foi realiza-lo. A escala ainda conta com a ilustração de carinhas, e cores para facilitar a sua avaliação.

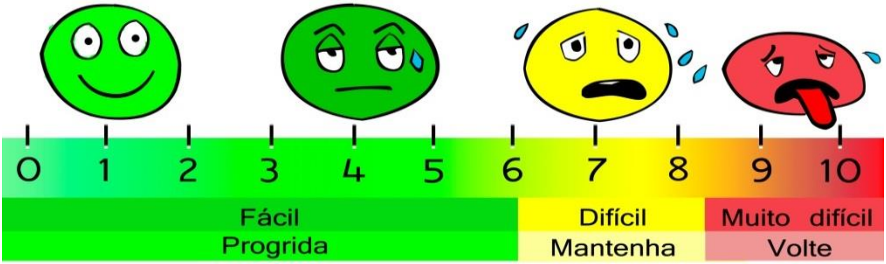

Fácil - Verde = Aumente a progressão

Caso, considere a execução de fácil a moderado, no dia seguinte deve aumentar o número de repetições.

Difícil - Amarelo – Mantenha a progressão

Se a realização dos exercícios for difícil ou cansativa, mantenha a última quantidade de repetições. Lembrando, só aumente quando se tornar fácil.

Muito difícil - Vermelho- Volte para a progressão anterior

Caso, considere a realização do exercício muito difícil, no dia seguinte deve voltar para a progressão da linha anterior.

Cada exercício deve ser realizado uma fez ao dia, pois objetivo é ir aumentando a progressão dos exercícios, conforme a sua facilidade.

No início, fazer os exercícios pode parecer difícil ou cansativo, mas é importante persistir, pois realizar essa atividade é uma maneira de diminuir alguns efeitos causados pela neuropatia diabética, que poderão dificultar a execução de atividades cotidianas, como caminhar.

É importante lembra que:

- O controle da glicemia é fundamental para prevenir o aparecimento ou a piora do Diabetes Mellitus.
- Essa cartilha não substitui o tratamento médico nem a necessidade de prática de atividade física regular! Continue tomando as medicações receitadas e compareça às consultas e exames agendados.

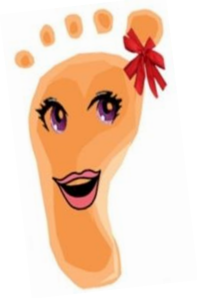

Se a dor for muito forte e persistir dia e noite, interrompa os exercícios e procure um médico o mais rápido possível! **Pode ser sinal de algum problema mais grave!**

Você possui outras dúvidas?

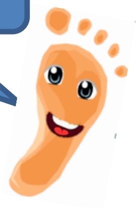

Os exercícios somente devem ser feitos durante 30 dias?

Não, os 30 dias são um período para se acostumar com prática diária desses exercícios, pois o ideal é que você os faça rotineiramente, ou seja, que sejam incorporados no seu dia-a-dia. Lembre-se que quem tem Diabetes tem a tendência de sofrer piora na condição dos pés se não os exercitar continuamente.

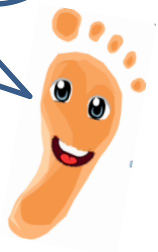

Quando eu já conseguir fazer os exercícios com a quantidade máxima de repetições, devo parar?

Não, você deve continuar a se exercitar mantendo ou aumentando a quantidade de repetições, conforme perceba que seja possível. Esperamos que você aprenda a conhecer os limites do seu corpo e que seja capaz de regular a intensidade sem exagerar!

Eu ainda não sei se posso fazer os exercícios. Será que eles irão me prejudicar?

Se você tiver dúvidas sobre o que estiver sentindo, se o que está fazendo é adequado, ou se estiver inseguro quanto à prática dos exercícios, consulte a equipe de saúde que o acompanha. É muito importante que você se informe para que a prática seja segura e que possa te trazer benefícios.

Em que lugar devo fazer os exercícios?

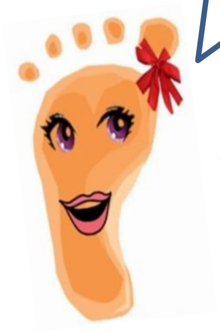

Você pode começar na sua própria casa, no seu trabalho, ou outro local em que seja possível prestar atenção nos movimentos que realiza. Assim irá aprender rapidamente.

Devo tomar algum cuidado específico?

O ideal é que faça em frente a um espelho, para enxergar bem a sua postura e se está fazendo os movimentos corretos. Quando fizer exercícios em pé, certifique-se de que tem onde se apoiar, como uma cadeira ou mesa bem fixas (que não balancem), uma parede ou outro local seguro. Não se exponha a situações em que poderá cair!

Preparação dos pés

COMO FAZER

1º Parte: Sentado, cruze uma perna sobre a outra. Massageie a planta dos pés com as duas mãos, **durante 1 minuto**. Faça movimentos circulares e de deslizamento com seus dedos. Depois realize o mesmo procedimento com o outro pé.

2º Parte: Coloque a bolinha no chão e deslize o pé sobre ela. Deixe a bolinha percorrer toda a planta dos pés, desde o calcanhar até a ponta dos dedos. Faça o movimento lentamente, procure sentir o contato da bolinha com sua pele, **durante 1 minuto cada pé.**

3º Parte: Sentado, cruze uma perna sobre a outra. Segure um dedo do pé por vez e gire cuidadosamente de um lado para o outro. Como se fosse um parafuso. Faça com todos os dedos dos pés, **10 vezes cada dedo.**

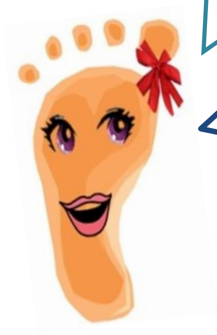

Esses movimentos devem ser feitos antes e depois da sessão de exercícios, como aquecimento e relaxamento. Podem ser feitos também após um dia de caminhada intensa, ou quando seus pés estiverem "cansados".

1º Parte

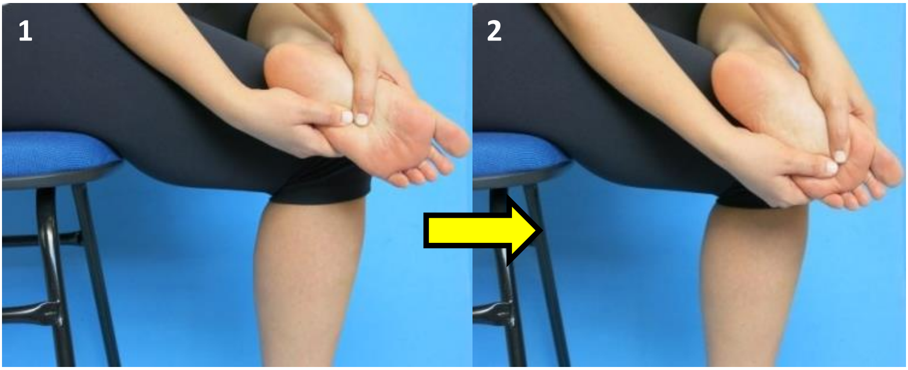

2º Parte

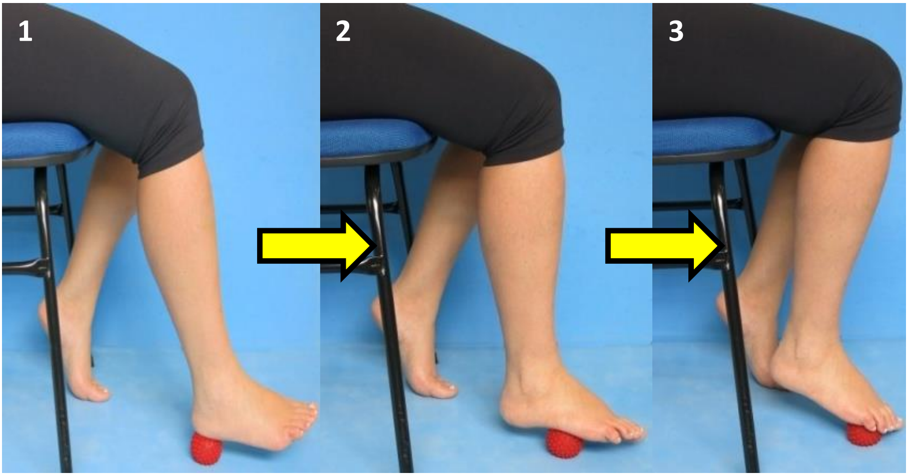

3º Parte

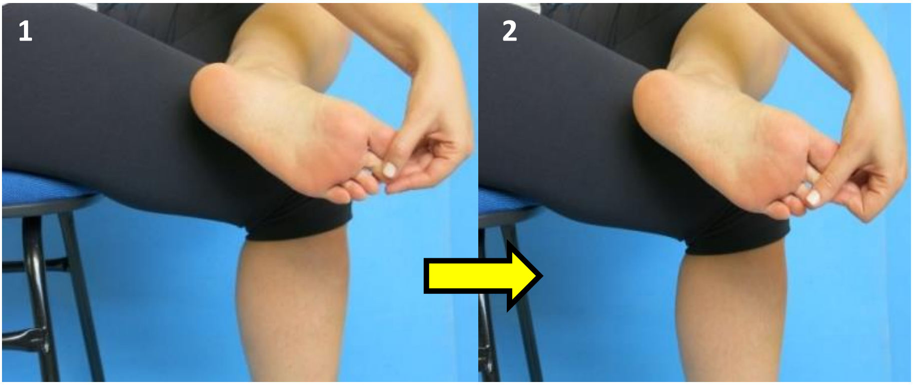

Exercício 1

Descrição

Eleve as bordas dos pés, alternadamente, sem tirar o dedo mínimo do chão com o pé esquerdo e sem tirar o dedão do chão com pé direito. Agora, inverta o procedimento: tente levantar os pés simultaneamente, mas sem tirar o dedinho do chão com o pé direito e sem tirar o dedão do chão com o pé esquerdo.

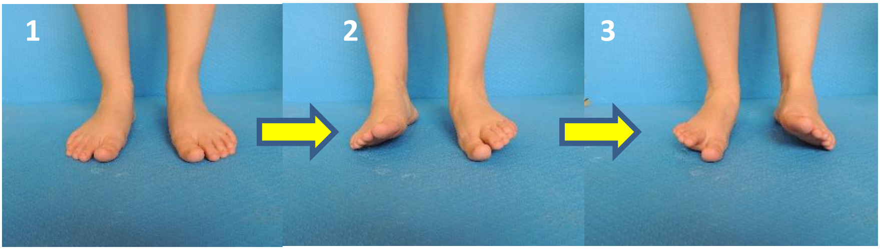

Comece em pé, com os dois pés apoiados. Apoie as mãos em uma cadeira ou mesa firme para manter-se equilibrado. Depois irá progredir para o apoio em um pé só.

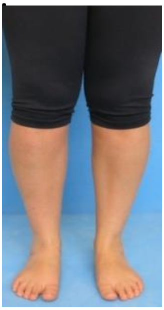

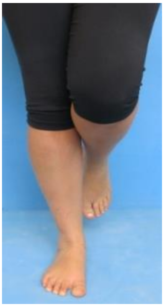

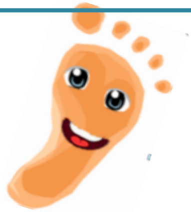

Como foi fazer os exercícios?

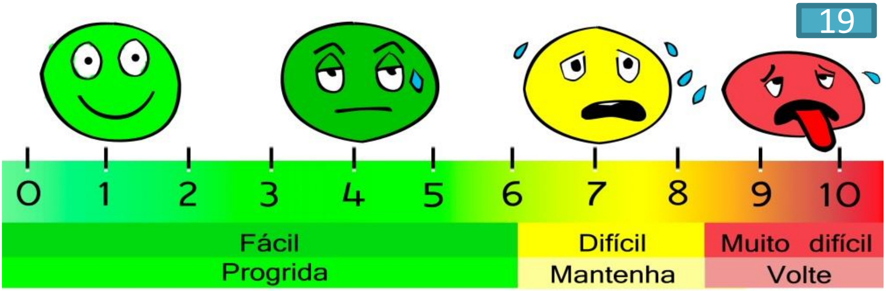

	Calendário da realização dos exercícios										
	**Dias**	1	2	3	4	5	6	7	8	9	10
	**Data**										
Sentado	30x										
	2x30										
Em pé	30x										
	2x30										
Sobre um pé	30x										
	2x30										
	**Dias**	11	12	13	14	15	16	17	18	19	20
	**Data**										
Sentado	30x										
	2x30										
Em pé	30x										
	2x30										
Sobre um pé	30x										
	2x30										
	**Dias**	21	22	23	24	25	26	27	28	29	30
	**Data**										
Sentado	30x										
	2x30										
Em pé	30x										
	2x30										
Sobre um pé	30x										
	2x30										

Exercício 2

Descrição

Com os pés bem apoiados no chão, abra e feche os dedos dos pés, sem tirar o calcanhar do chão. Faça o movimento devagar e tente afastá-los o máximo que conseguir.

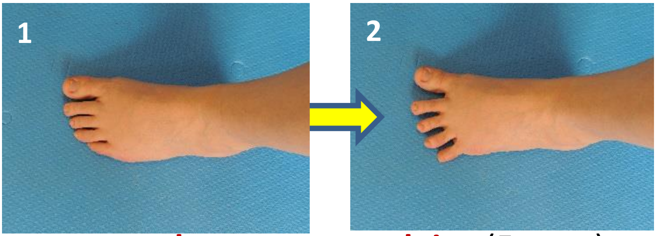

Comece sentado em uma cadeira (Foto a), e faça um pé de cada vez. Depois que tiver aprendido, faça com os dois pés ao mesmo tempo (Foto b). Com a melhora, faça o mesmo em pé e depois apoiando em um pé só (Foto c).

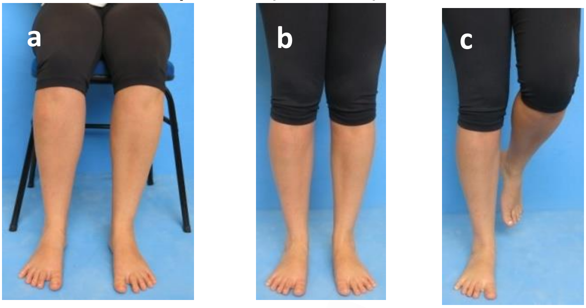

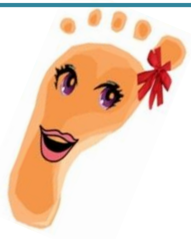

Como foi fazer os exercícios?

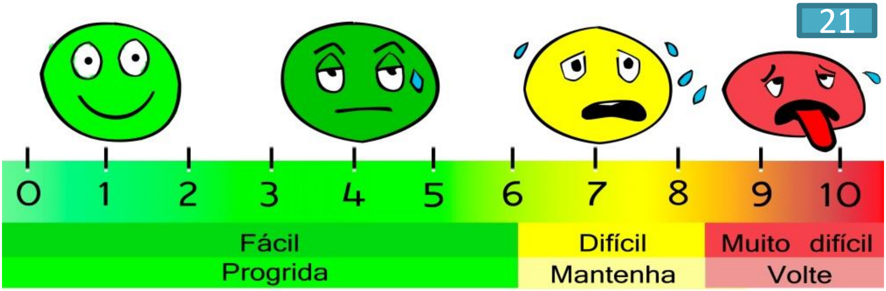

Calendário da realização dos exercícios

	Dias	1	2	3	4	5	6	7	8	9	10
	Data										
Sentado	30x										
	2x30										
Em pé	30x										
	2x30										
Sobre um pé	30x										
	2x30										
	Dias	11	12	13	14	15	16	17	18	19	20
	Data										
Sentado	30x										
	2x30										
Em pé	30x										
	2x30										
Sobre um pé	30x										
	2x30										
	Dias	21	22	23	24	25	26	27	28	29	30
	Data										
Sentado	30x										
	2x30										
Em pé	30x										
	2x30										
Sobre um pé	30x										
	2x30										

Exercício 3

Descrição

Encoste, alternadamente, o dedão e o dedinho do pé no chão, mas sem tirar o calcanhar do chão. Faça devagar para controlar bem o movimento, se possível com os pés direito e esquerdo ao mesmo tempo (Foto 1 e 2).

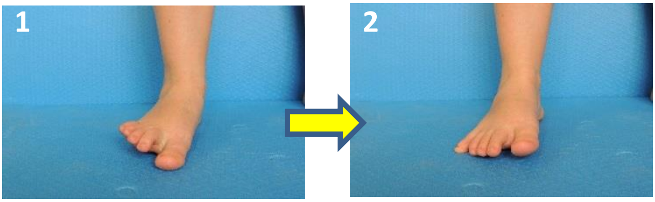

Comece sentado em uma cadeira, e faça um pé de cada vez (Foto a). Depois que tiver aprendido, faça com os dois pés ao mesmo tempo (Foto b). Depois progrida para fazer o mesmo em pé e depois apoiando em um pé só (Foto c).

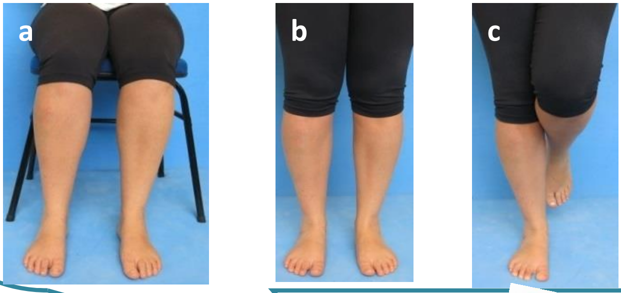

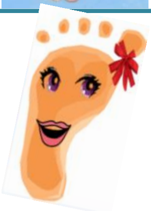

Como foi fazer os exercícios?

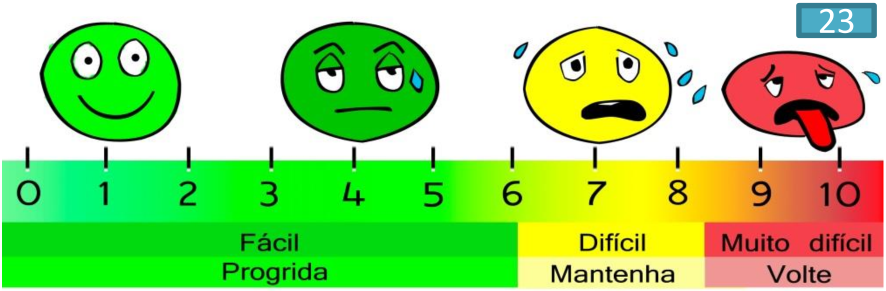

	Calendário da realização dos exercícios										
	Dias	1	2	3	4	5	6	7	8	9	10
	Data										
Sentado	30x										
	2x30										
Em pé	30x										
	2x30										
Sobre um pé	30x										
	2x30										
	Dias	11	12	13	14	15	16	17	18	19	20
	Data										
Sentado	30x										
	2x30										
Em pé	30x										
	2x30										
Sobre um pé	30x										
	2x30										
	Dias	21	22	23	24	25	26	27	28	29	30
	Data										
Sentado	30x										
	2x30										
Em pé	30x										
	2x30										
Sobre um pé	30x										
	2x30										

Exercício 4

DESCRIÇÃO

Pegue um objeto com os dedos dos pés (Foto 1 e 2), levante-o do chão (Foto 3) e solte-o (Foto 4).

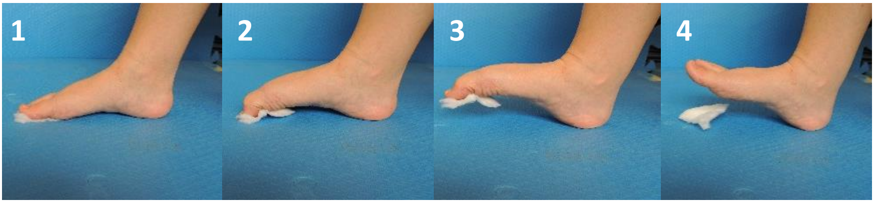

Realizar o mesmo exercício sempre com um pé de cada vez (Foto a).

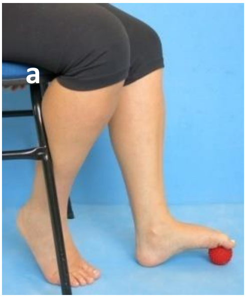

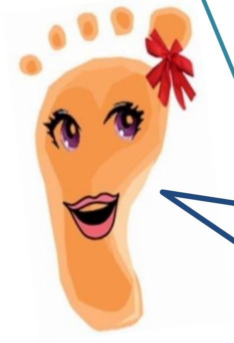

Notou que a tabela desse exercício é um pouco diferente? Além de aumentar a progressão você também deve mudar o material de realização do exercício.

Como foi fazer os exercícios?

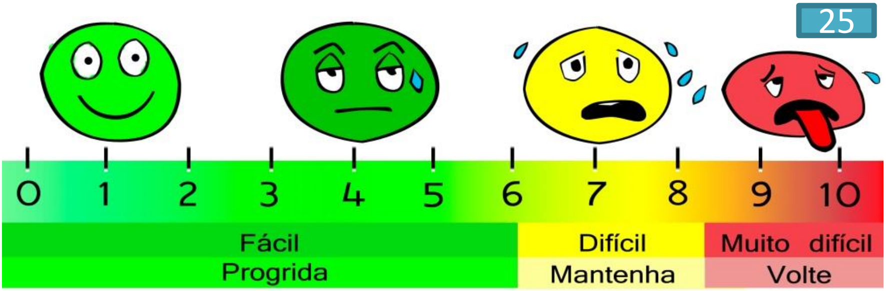

	Calendário da realização dos exercícios										
	Dias	1	2	3	4	5	6	7	8	9	10
	Data										
Lápis	30x										
	2x30										
Bolinha	30x										
	2x30										
Algodão	30x										
	2x30										
	Dias	11	12	13	14	15	16	17	18	19	20
	Data										
Lápis	30x										
	2x30										
Bolinha	30x										
	2x30										
Algodão	30x										
	2x30										
	Dias	21	22	23	24	25	26	27	28	29	30
	Data										
Lápis	30x										
	2x30										
Bolinha	30x										
	2x30										
Algodão	30x										
	2x30										

Exercício 5

Descrição

Bater a região anterior do pé no chão, o mais rápido que conseguir, como se estivesse impaciente (Fotos 1, 2 e 3).

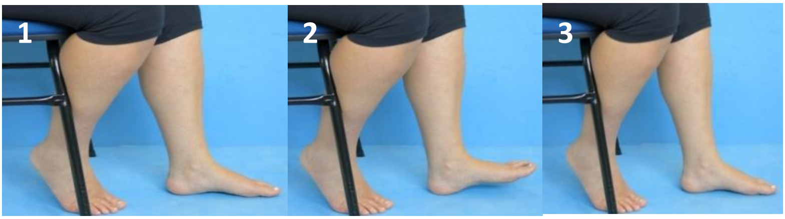

Comece sentado em uma cadeira, e faça um pé de cada vez (Foto a). Depois que tiver aprendido, faça o mesmo em pé (Foto a).

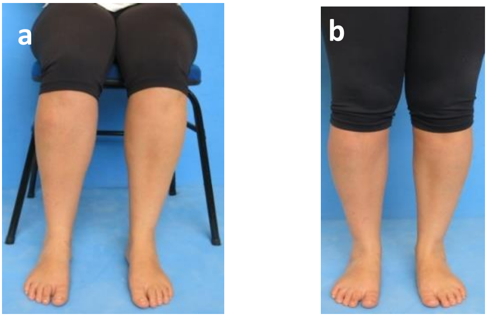

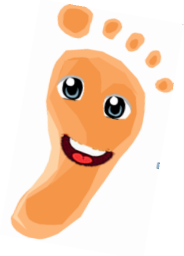

Como foi fazer os exercícios?

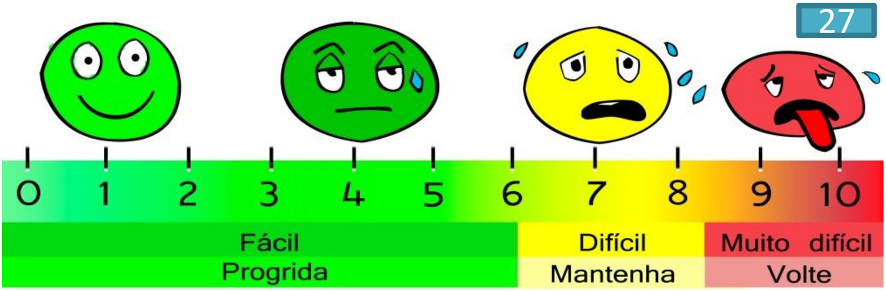

Calendário da realização dos exercícios

	Dias	1	2	3	4	5	6	7	8	9	10
	Data										
Sentado	30x										
	2x30										
Em pé	30x										
	2x30										
Sobre um pé	30x										
	2x30										
	Dias	11	12	13	14	15	16	17	18	19	20
	Data										
Sentado	30x										
	2x30										
Em pé	30x										
	2x30										
Sobre um pé	30x										
	2x30										
	Dias	21	22	23	24	25	26	27	28	29	30
	Data										
Sentado	30x										
	2x30										
Em pé	30x										
	2x30										
Sobre um pé	30x										
	2x30										

Exercício 6

DESCRIÇÃO

Comece em pé, com os dois pés no chão, apoie as mãos em uma cadeira ou mesa firme para manter-se equilibrado, depois suba e desça nas pontas dos pés (Foto 1 e 2).

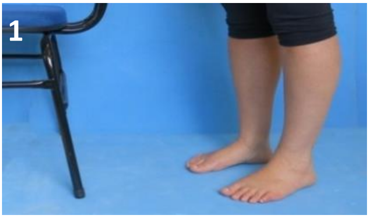

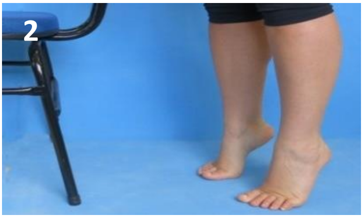

Depois irá progredir para o apoio em um pé só (Foto b).

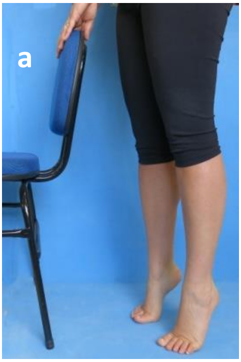

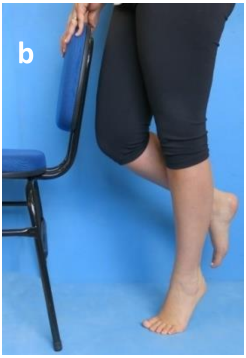

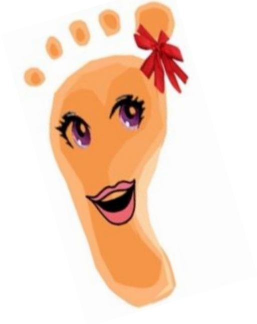

Como foi fazer os exercícios?

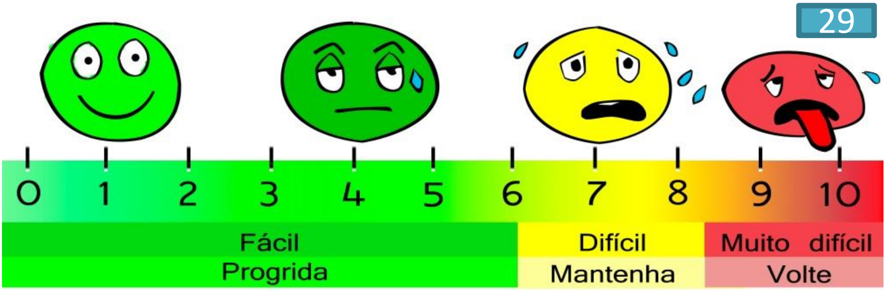

	Calendário da realização dos exercícios										
	Dias	1	2	3	4	5	6	7	8	9	10
	Data										
Sentado	30x										
	2x30										
Em pé	30x										
	2x30										
Sobre um pé	30x										
	2x30										
	Dias	11	12	13	14	15	16	17	18	19	20
	Data										
Sentado	30x										
	2x30										
Em pé	30x										
	2x30										
Sobre um pé	30x										
	2x30										
	Dias	21	22	23	24	25	26	27	28	29	30
	Data										
Sentado	30x										
	2x30										
Em pé	30x										
	2x30										
Sobre um pé	30x										
	2x30										

O que fazer quando terminar a tabela?

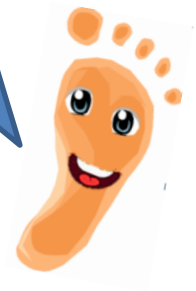

- **Continue fazendo os exercícios no seu dia-a-dia. Objetivo da cartilha é que após esses 30 dias, já tenha aprendido a importância e tornado um hábito o cuidado com os pés.**

- Caso não consiga realizar os exercícios todos os dias, realize de 3 a 4 vezes por semana.

- **Sempre observe os seus pés** para identificar se estão surgindo alguma ferida ou mudanças. Os principais cuidados estão explicados na próxima página.

Alguns cuidados que devemos ter com os pés

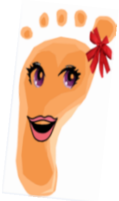

Se passa muito tempo sentado, evite manter as pernas cruzadas e procure mexer os pés a cada 30 minutos, para facilitar a circulação do sangue.

Meia

- Escolhas as meias sem costura e sem elástico, pois evitam machucados.

Unhas

- O corte deve ser quadrado, deixando ver uma pequena parte branca, para evitar que sua unha encrave.
- Preferencialmente, consulte um podólogo. Se você utilizar os serviços de manicures e pedicures Informe sempre que é uma pessoa com diabetes e peça para que não tirem as cutículas e não cortar os cantos das unhas. **Caso suas unhas estejam encravadas, muito grossas, com aspecto “esfarelado”, com alterações na cor ou apresente qualquer outra alteração, procure o serviço de saúde mais próximo de sua casa.**

Calçados

- Evite andar descalço, mesmo dentro de casa.
- Verifique o interior dos sapatos procurando deformidades nas palmilhas ou costuras, para evitar feridas nos pés .
- Evite usar sapatos sem meia
- Guarde seus sapatos em ambiente arejado e lave-os sempre que necessário. Deixe secar bem antes de usá-los.
- Os chinelos de dedo ou tiras não protegem os pés, e devem ser evitados.

Existem calçados e palmilhas específicos para pessoa com diabetes. Procure a Unidade Básica de Saúde (Posto de Saúde) mais próxima de sua residência e faça uma consulta com um médico do Programa de Saúde da Família (PSF) ou credenciado pelo SUS, e havendo indicação, ele irá prescrever o sapato para você.

Aos profissionais da saúde

Esse material pode ser utilizado para orientar um **grupo** de pessoas com diabetes **em atenção primária**, em atendimentos **domiciliares** ou até mesmo em atendimentos **individuais**. No contexto hospitalar, é provável que sejam necessárias adaptações, que devem ser discutidas com a equipe de saúde.

O profissional da área da saúde não precisa ter um conhecimento especifico sobre DM, mas precisa conhecer muito bem o material.

Importante lembrar que :

A abordagem do membro inferior da pessoa com diabetes não é desvinculada dos cuidados gerais (controle da glicemia, hipertensão, obesidade, tabagismo, atividade física e alimentação) que são decisivos para melhorar a qualidade de vida do usuário.

Todas as pessoas com diabetes devem ter seus pés examinados nas consultas.

Aos profissionais da saúde

- Ao apresentar o material, faça a leitura do material junto com as pessoas;
- Explique o exercício e o realize junto para demonstrar. Se necessário repita o procedimento;
- O aprendizado está a associado à prática, então, sempre que possível, peça para repetirem os exercícios em sua presença de modo a corrigir possíveis alterações;
- Para quem apresente muita dificuldade na realização ou na compreensão dos exercícios, instrua um exercício novo por atendimento;
- Empodere os cuidadores: explique a importância de cuidar dos pés e informe que pode fazer os exercícios em conjunto como incentivo.

Aos profissionais da saúde

•Caso deseje aplicar esses exercícios em um GRUPO, FIQUE ATENTO para o seguinte:

- Durante a realização dos exercícios, aproxime-se de cada participante e os observe individualmente;
- Lembre-se de não comparar os participantes do grupo. Cada um tem o seu próprio processo de aprendizado e suas capacidades físicas individuais.

Para mais informações sobre o grupo de pesquisa que desenvolveu este material, acesse o site:

http://www.usp.br/labimph

Para classificar o grau da neuropatia diabética, está disponível gratuitamente um software fuzzy, criado para o uso de profissionais da saúde no site:

http://www.usp.br/labimph/fuzzy/.

LaBiMPH USP

Laboratório de Biomecânica do Movimento e Postura Humana

USP

Universidade de São Paulo

Laboratório de Biomecânica do Movimento e da Postura Humana

www.usp.br/labimph
